# Supplementary material for: An In Vitro Antimicrobial, Anticancer and Antioxidant Activity of N–[(2–Arylmethylthio)phenylsulfonyl]cinnamamide Derivatives
Source: Molecules. 2023 Mar 30;28(7):3087. doi: 10.3390/molecules28073087 (PMC10096175; doi:10.3390/molecules28073087)
Supplement: Supplementary file 1 [file molecules-28-03087-s001.zip › molecules-2289284-SI.pdf]

Supplementary material

An In Vitro Antimicrobial, Anticancer and Antioxidant Activity of *N*–[(2–Arylmethylthio)phenylsulfonyl]cinnamamide Derivatives

Anita Bułakowska [1,*,†], Jarosław Sławiński [1], Rafał Hałasa [2,†], Anna Hering [3], Magdalena Gucwa [3], J. Renata Ochocka [3] and Justyna Stefanowicz-Hajduk [3,*]

[1] Department of Organic Chemistry, Medical University of Gdańsk, Al. Gen. J. Hallera 107, 80-416 Gdansk, Poland; jaroslaw@gumed.edu.pl
[2] Department of Pharmaceutical Microbiology, Medical University of Gdańsk, Al. Gen. J. Hallera 107, 80-416 Gdansk, Poland; rafal.halasa@gumed.edu.pl
[3] Department of Biology and Pharmaceutical Botany, Medical University of Gdańsk, Al. Gen. J. Hallera 107, 80-416 Gdansk, Poland; anna.hering@gumed.edu.pl (A.H.); magdalena.gucwa@gumed.edu.pl (M.G.); jadwiga.ochocka@gumed.edu.pl (J.R.O.)
* Correspondence: bulanit@gumed.edu.pl (A.B.); justyna.stefanowicz-hajduk@gumed.edu.pl (J.S.-H.)
† These authors contributed equally to this work.

Tabele of contents

Appendix A:

Spectrum 1. ^{1}H NMR of compd **2** (500 MHz, DMSO-d_6).

Spectrum 2. ^{1}H NMR of compd **3** (500 MHz, DMSO-d_6).

Spectrum 3. ^{1}H NMR of compd **16a** (500 MHz, DMSO-d_6)

Spectrum 4. ^{1}H NMR of compd **16b** (500 MHz, DMSO-d_6).

Spectrum 5. ^{1}H NMR of compd **16c** (500 MHz, DMSO-d_6).

Spectrum 6. ^{1}H NMR of compd **16d** (500 MHz, DMSO-d_6)

Spectrum 7. ^{1}H NMR of compd **16e** (500 MHz, DMSO-d_6).

Spectrum 8. ^{13}C NMR of compd **16e** (125 MHz, DMSO-d_6).

Spectrum 9. ^{1}H NMR of compd **16f** (500 MHz, DMSO-d_6).

Spectrum 10. ^{13}C NMR of compd **16f** (125 MHz, DMSO-d_6).

Spectrum 11. ^{1}H NMR of compd **17a** (500 MHz, DMSO-d_6).

Spectrum 12. ^{13}C NMR of compd **17a** (125 MHz, DMSO-d_6).

Spectrum 13. ^{1}H NMR of compd **17b** (500 MHz, DMSO-d_6).

Spectrum 14. ^{13}C NMR of compd **17b** (125 MHz, DMSO-d_6).

Spectrum 15. ^{1}H NMR of compd **17c** (500 MHz, DMSO-d_6).

Spectrum 16. ^{13}C NMR of compd **17c** (125 MHz, DMSO-d_6).

Spectrum 17. ^{1}H NMR of compd **17d** (500 MHz, DMSO-d_6).

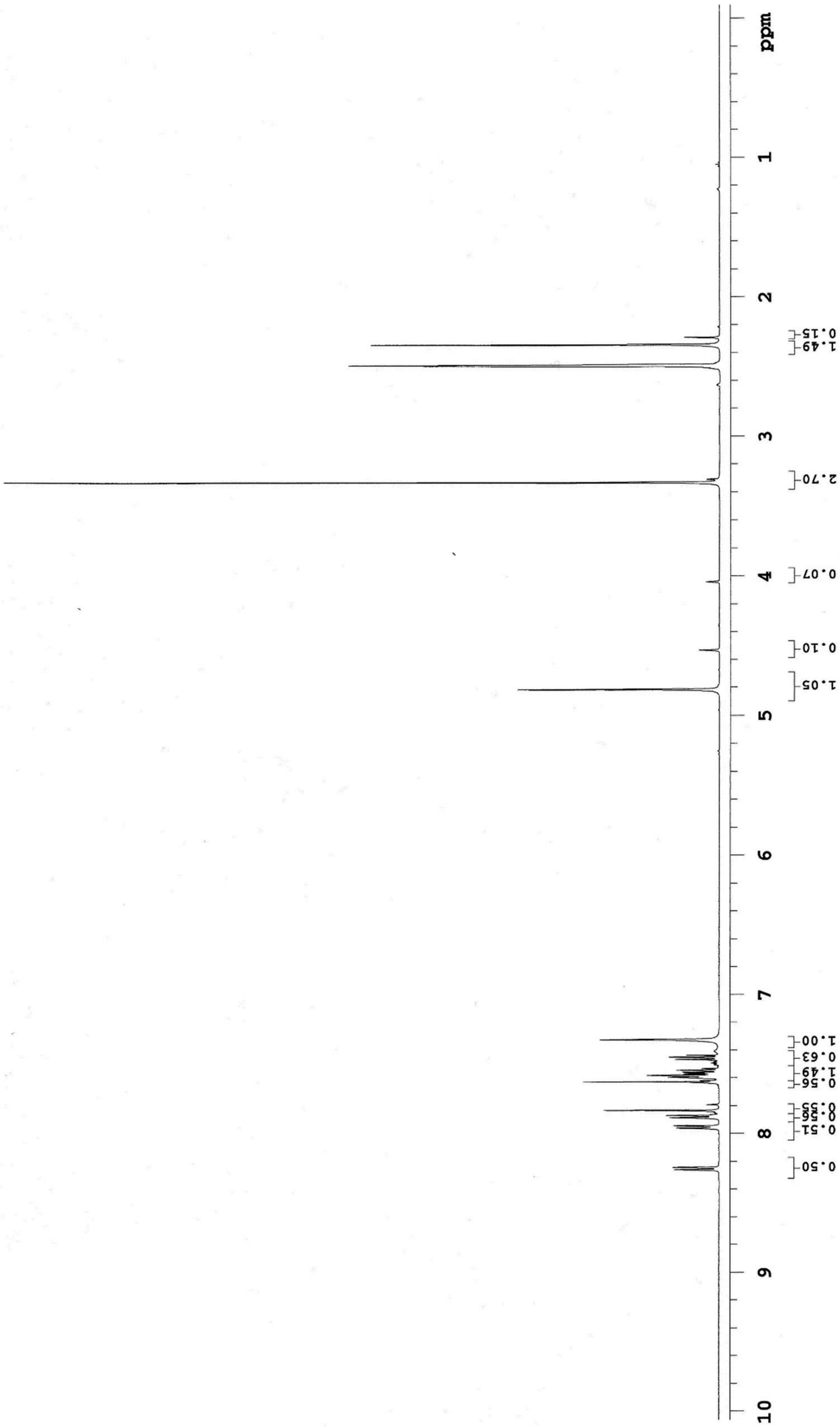

Spectrum 1. ^{1}H NMR of compd **2** (500 MHz, DMSO-d_6).

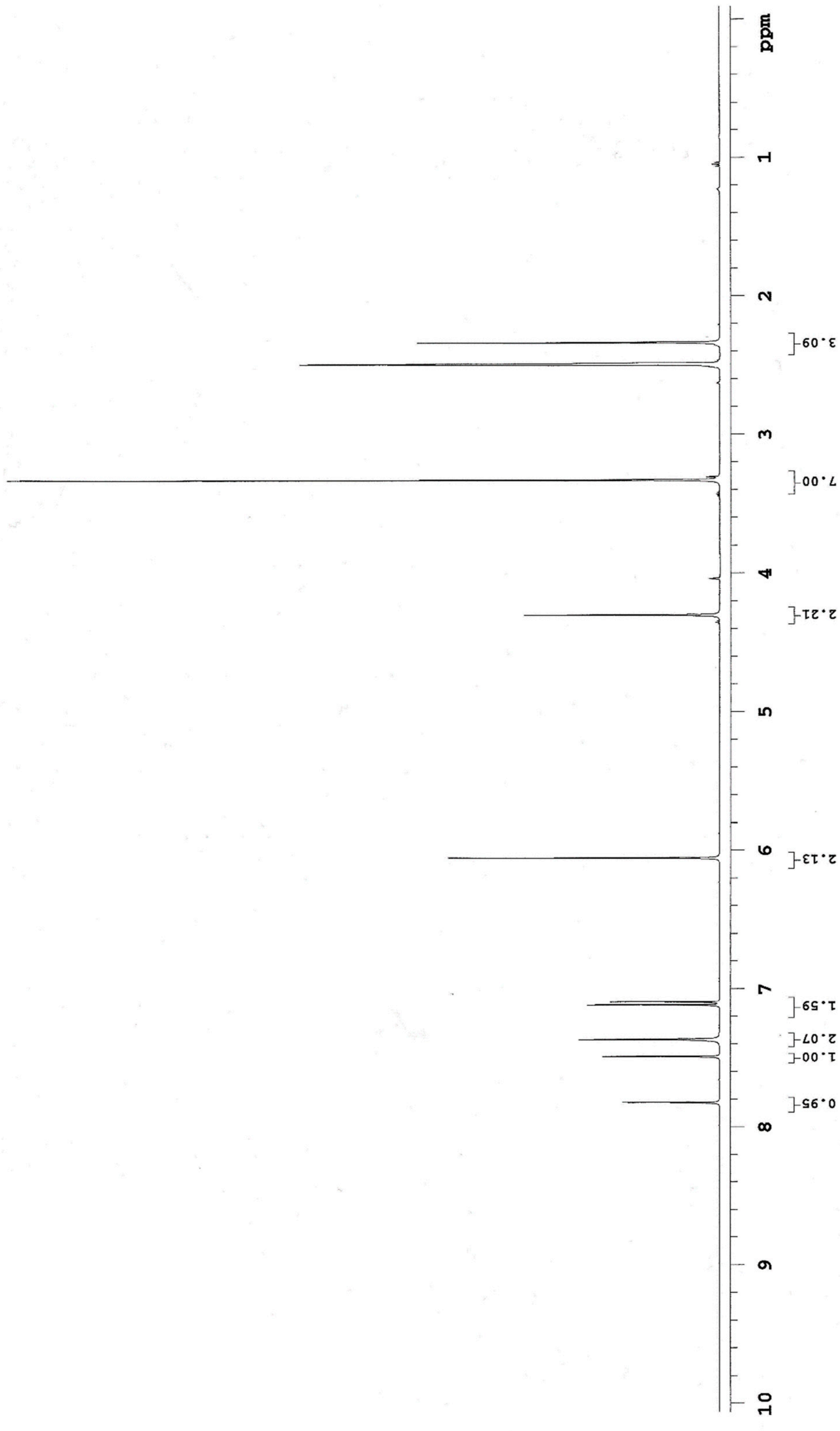

Spectrum 2. ^{1}H NMR of compd **3** (500 MHz, DMSO-d_6).

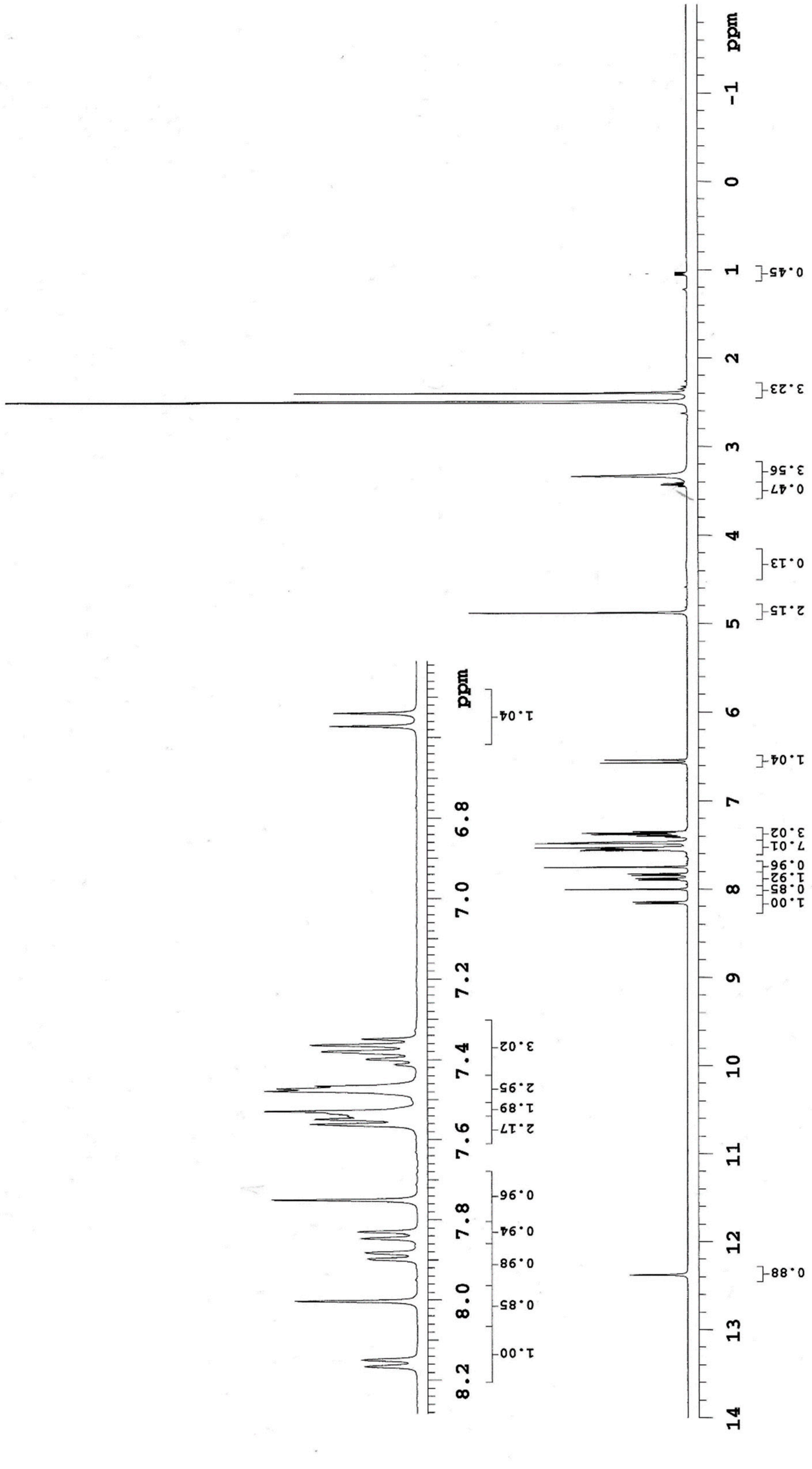

Spectrum 3. ^{1}H NMR of compd **16a** (500 MHz, DMSO-d_6).

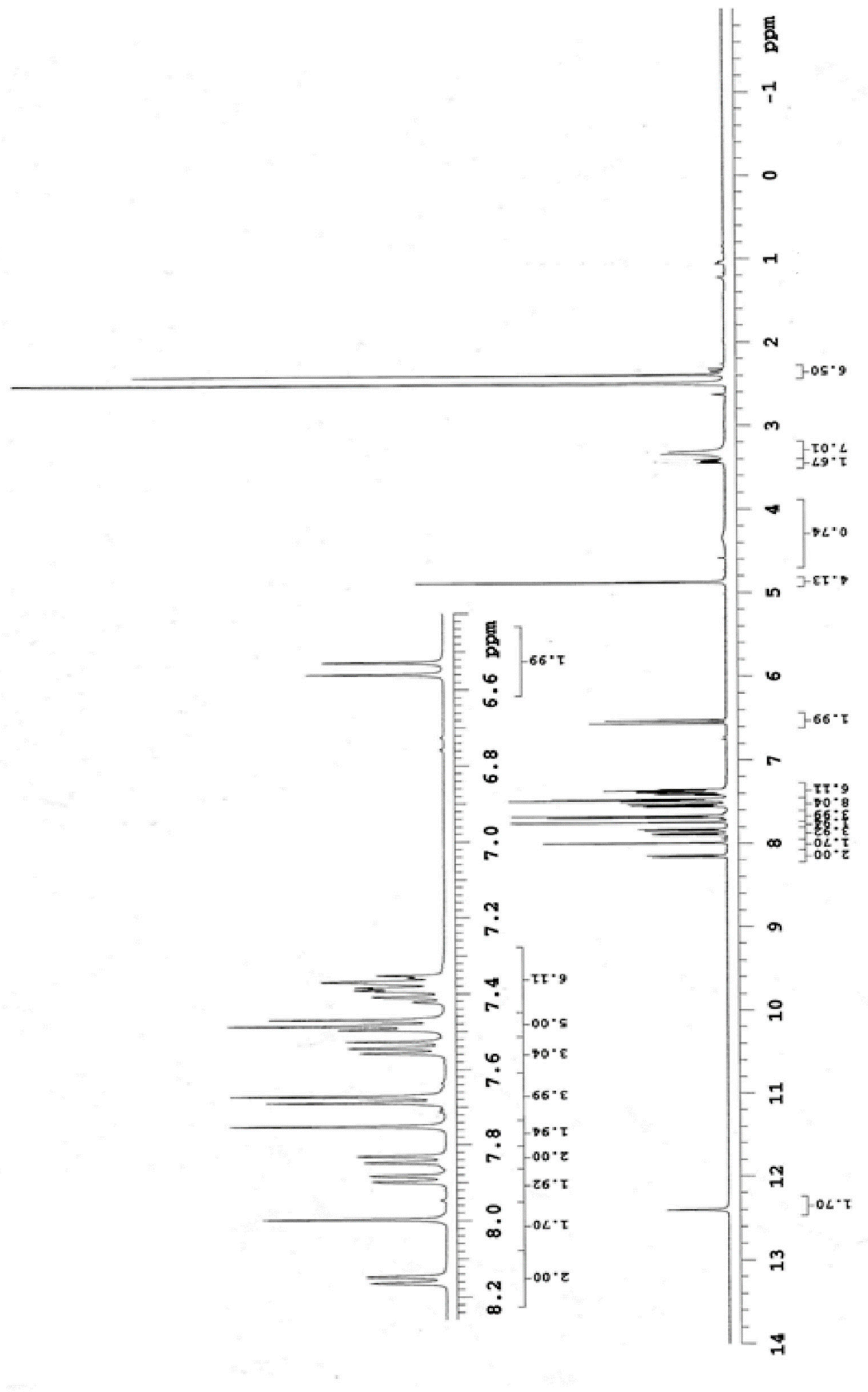

Spectrum 4. ^{1}H NMR of compd **16b** (500 MHz, DMSO-d_6).

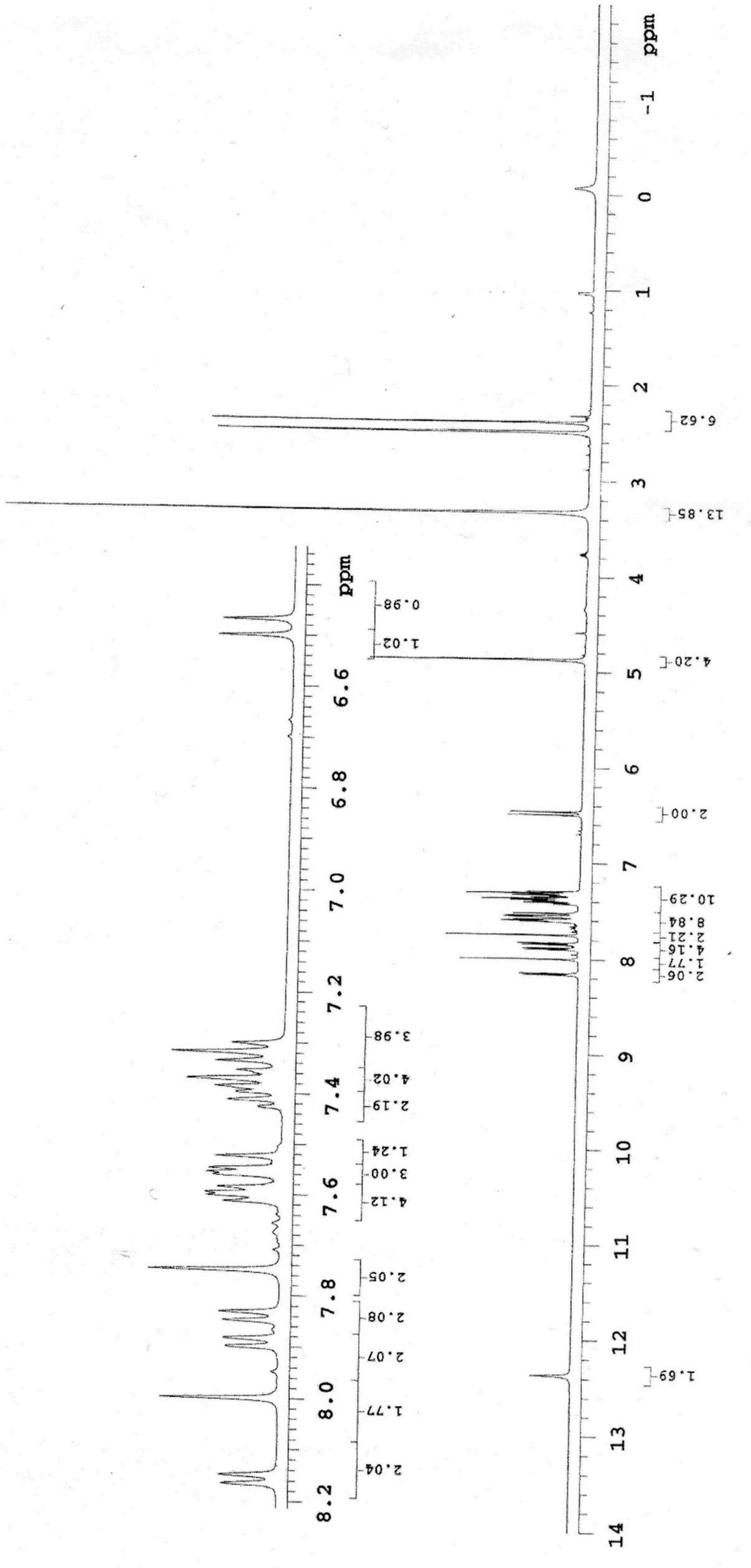

Spectrum 5. ^{1}H NMR of compd **16c** (500 MHz, DMSO-d_6).

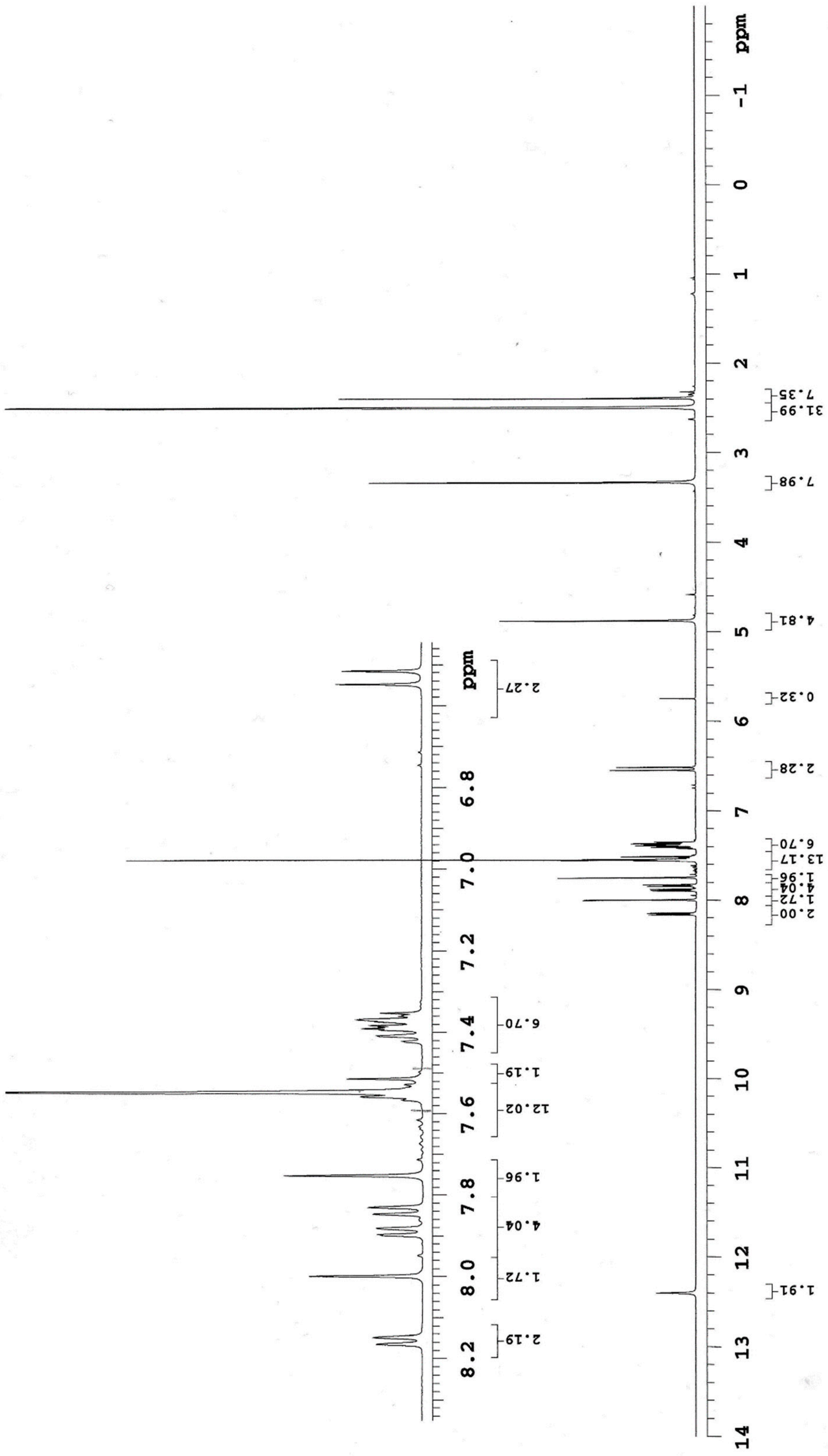

Spectrum 6. ^{1}H NMR of compd **16d** (500 MHz, DMSO-d_6).

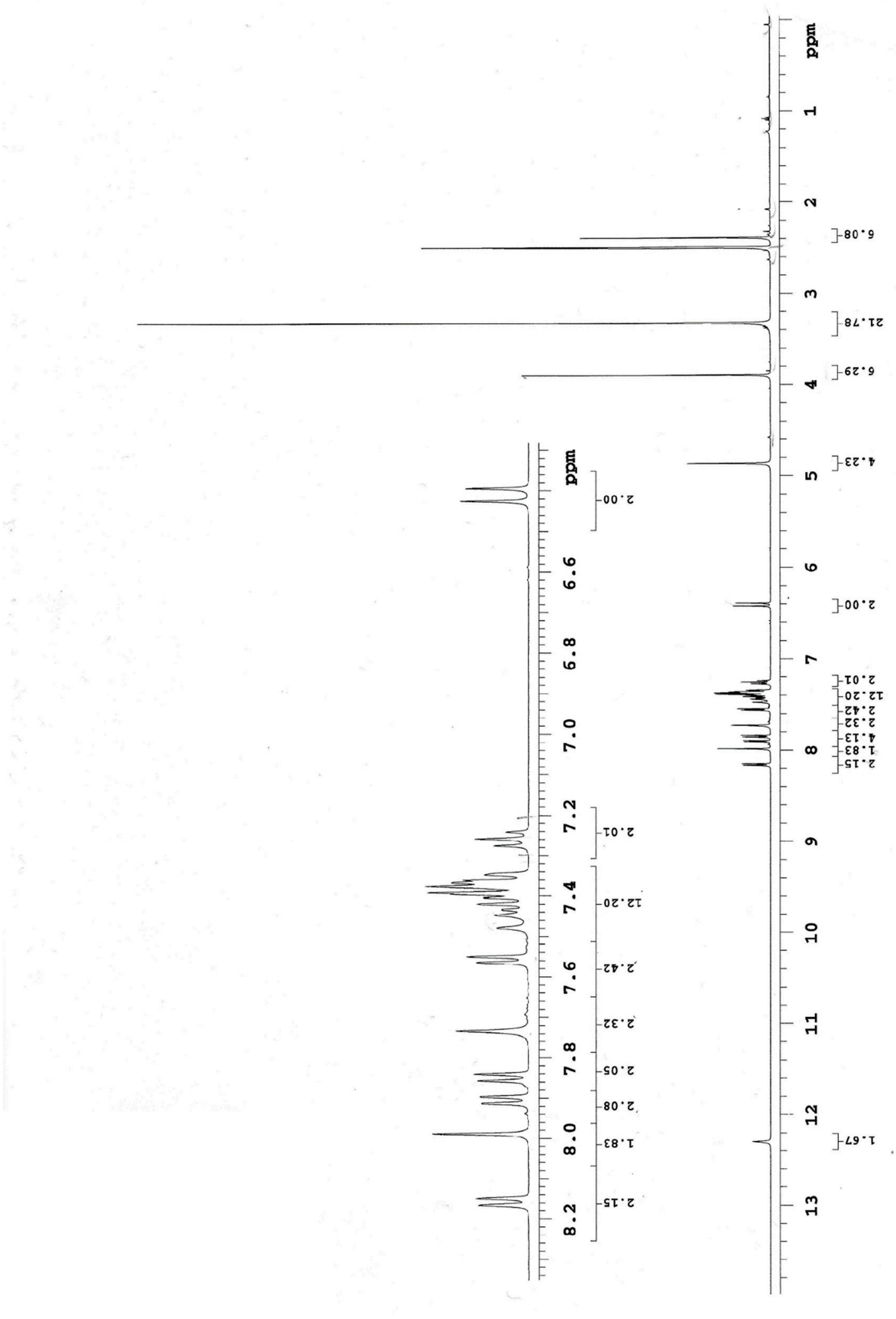

Spectrum 7. ^{1}H NMR of compd **16e** (500 MHz, DMSO-d_6).

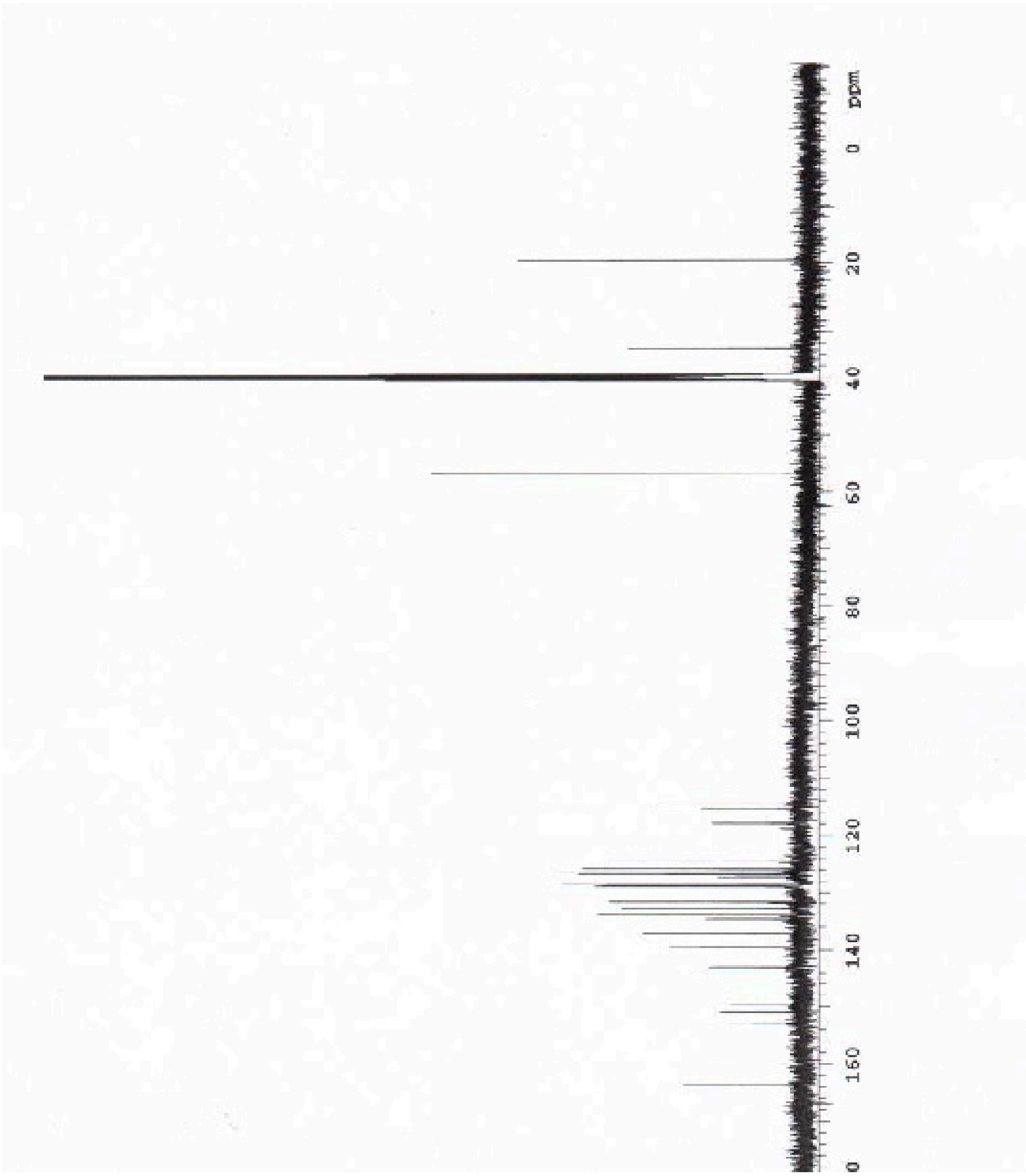

Spectrum 8. ^{13}C NMR of compd **16e** (125 MHz, DMSO-d_6).

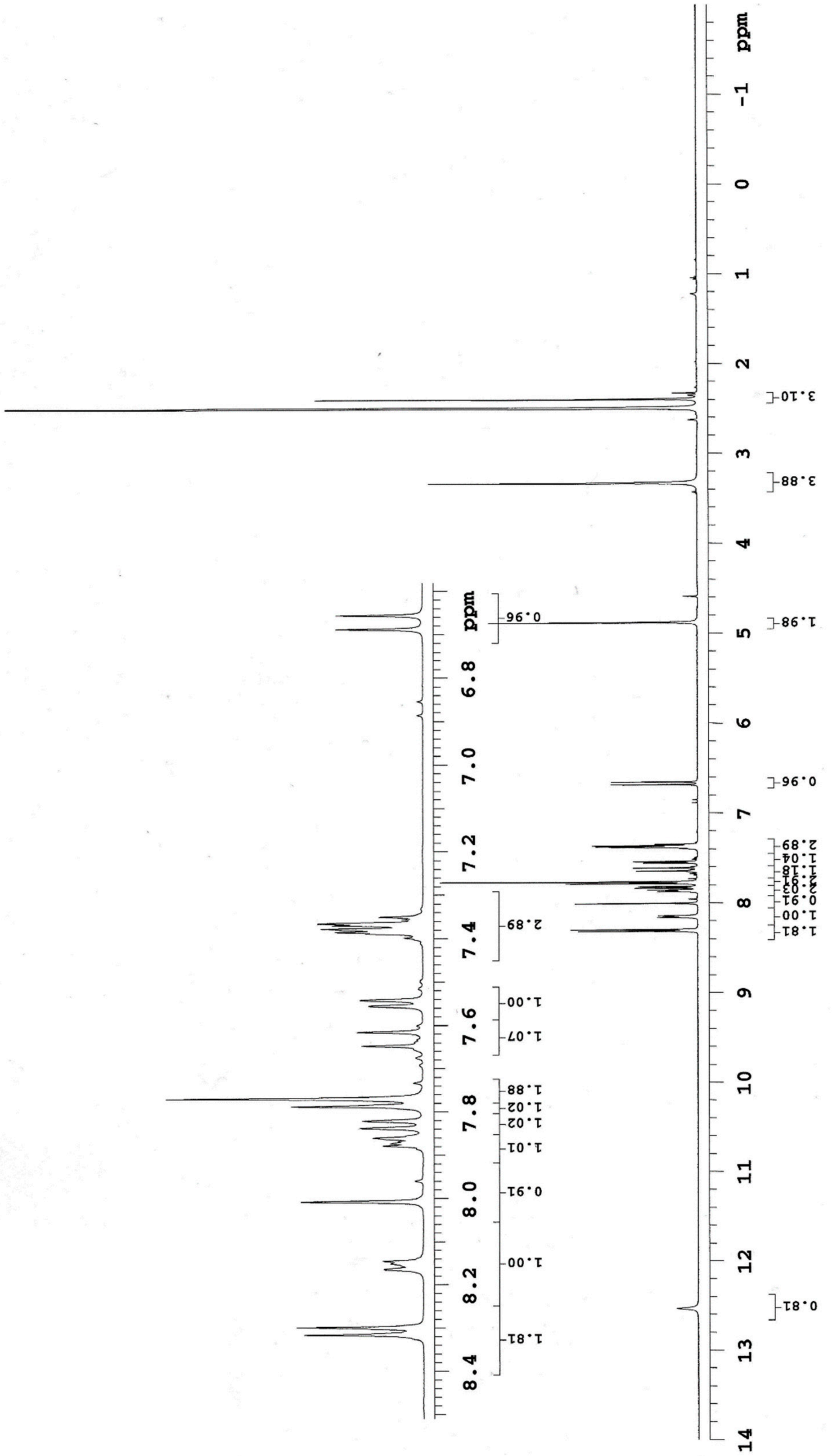

Spectrum 9. ^{1}H NMR of compd **16f** (500 MHz, DMSO-d_6).

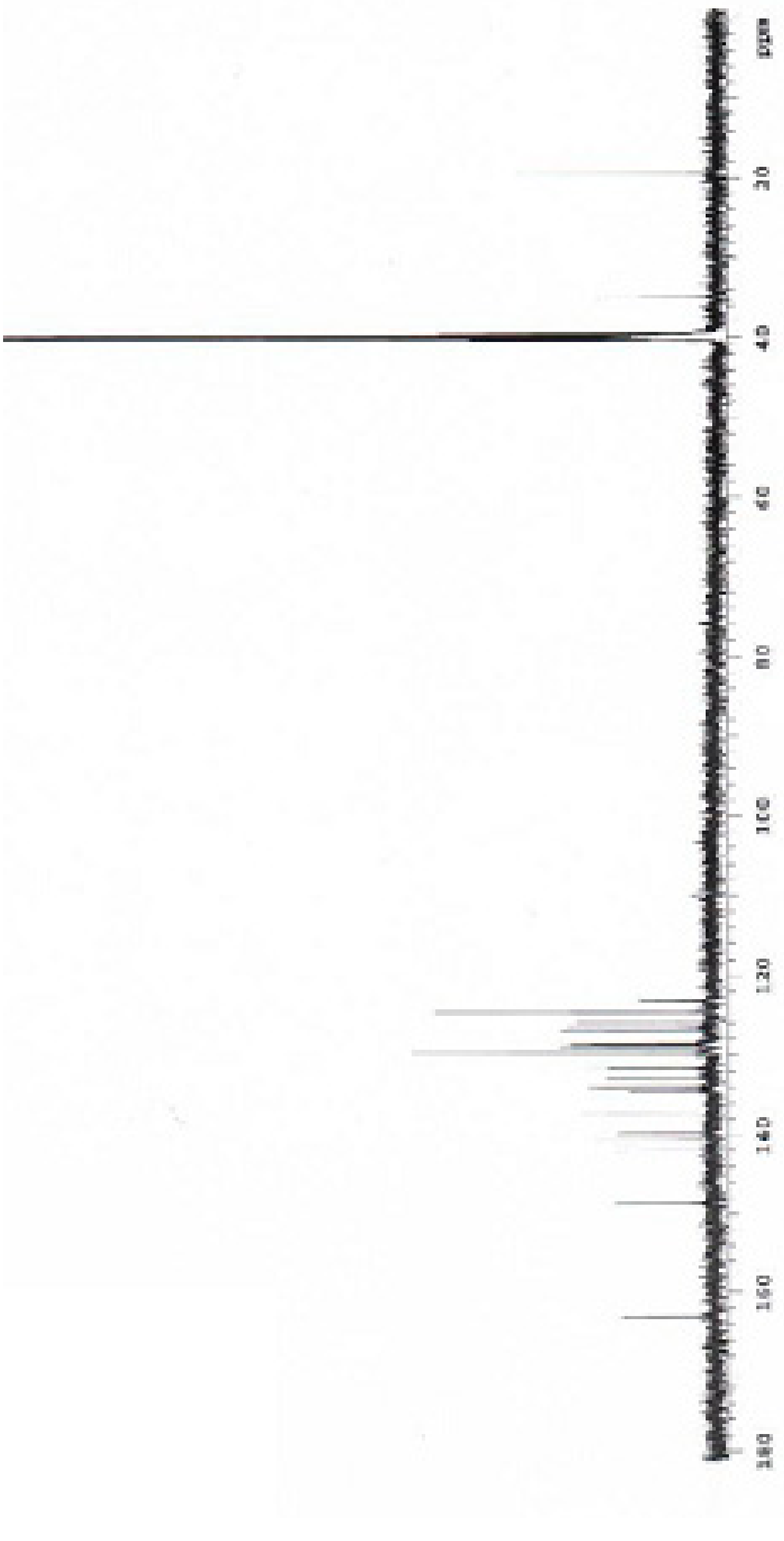

Spectrum 10. ^{13}C NMR of compd **16f** (125 MHz, DMSO-d_6).

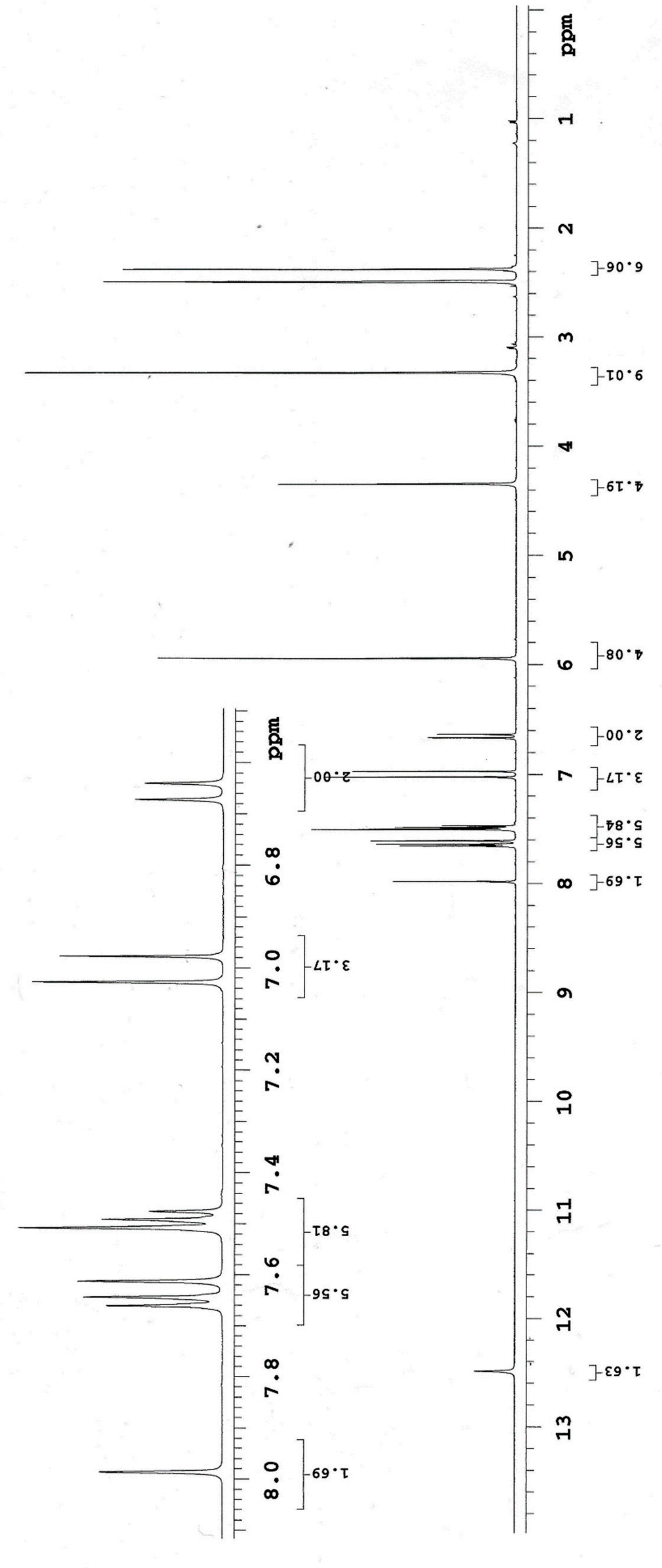

Spectrum 11. ^{1}H NMR of compd **17a** (500 MHz, DMSO-d_6).

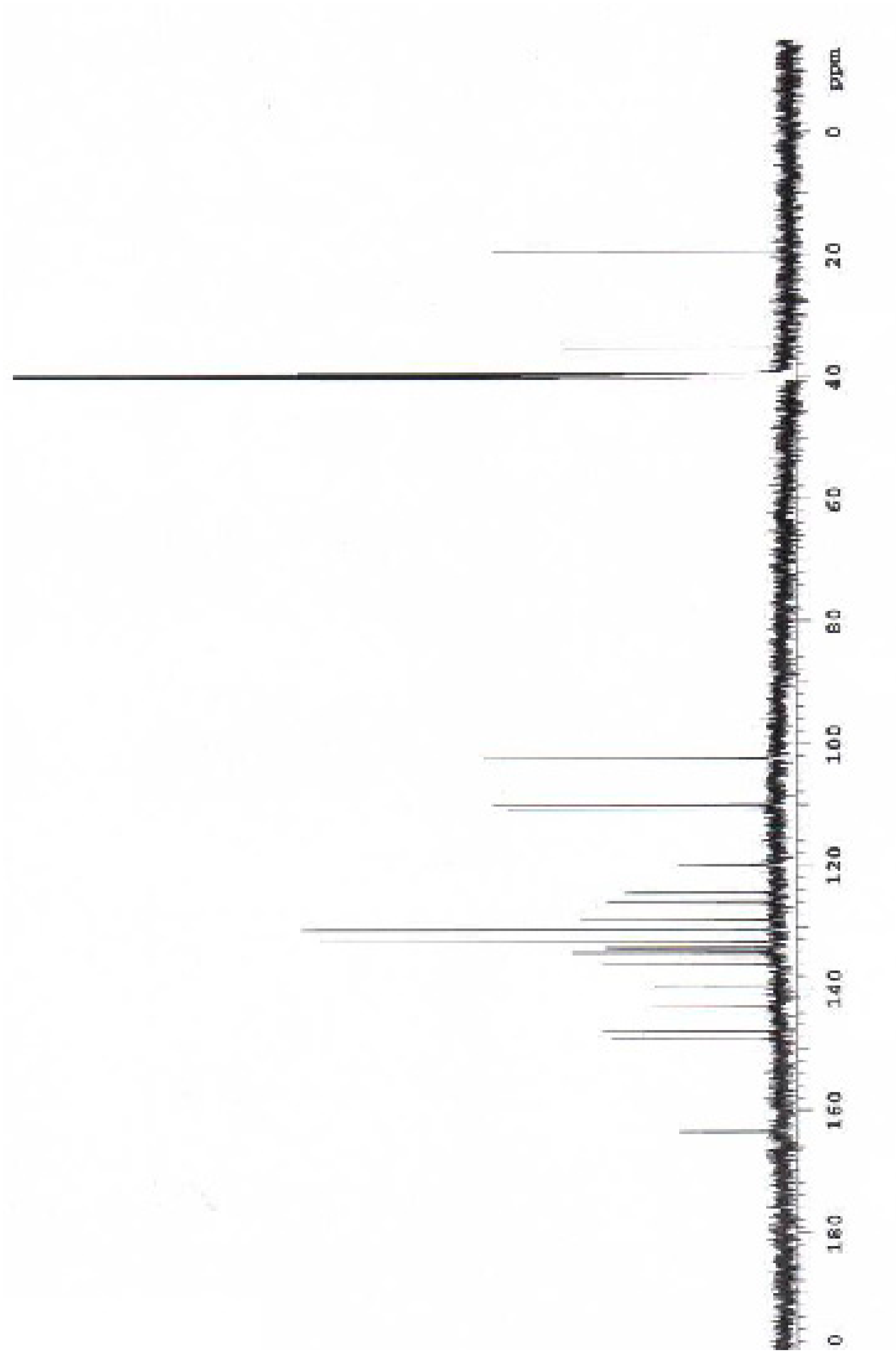

Spectrum 12. ^{13}C NMR of compd **17a** (125 MHz, DMSO-d_6).

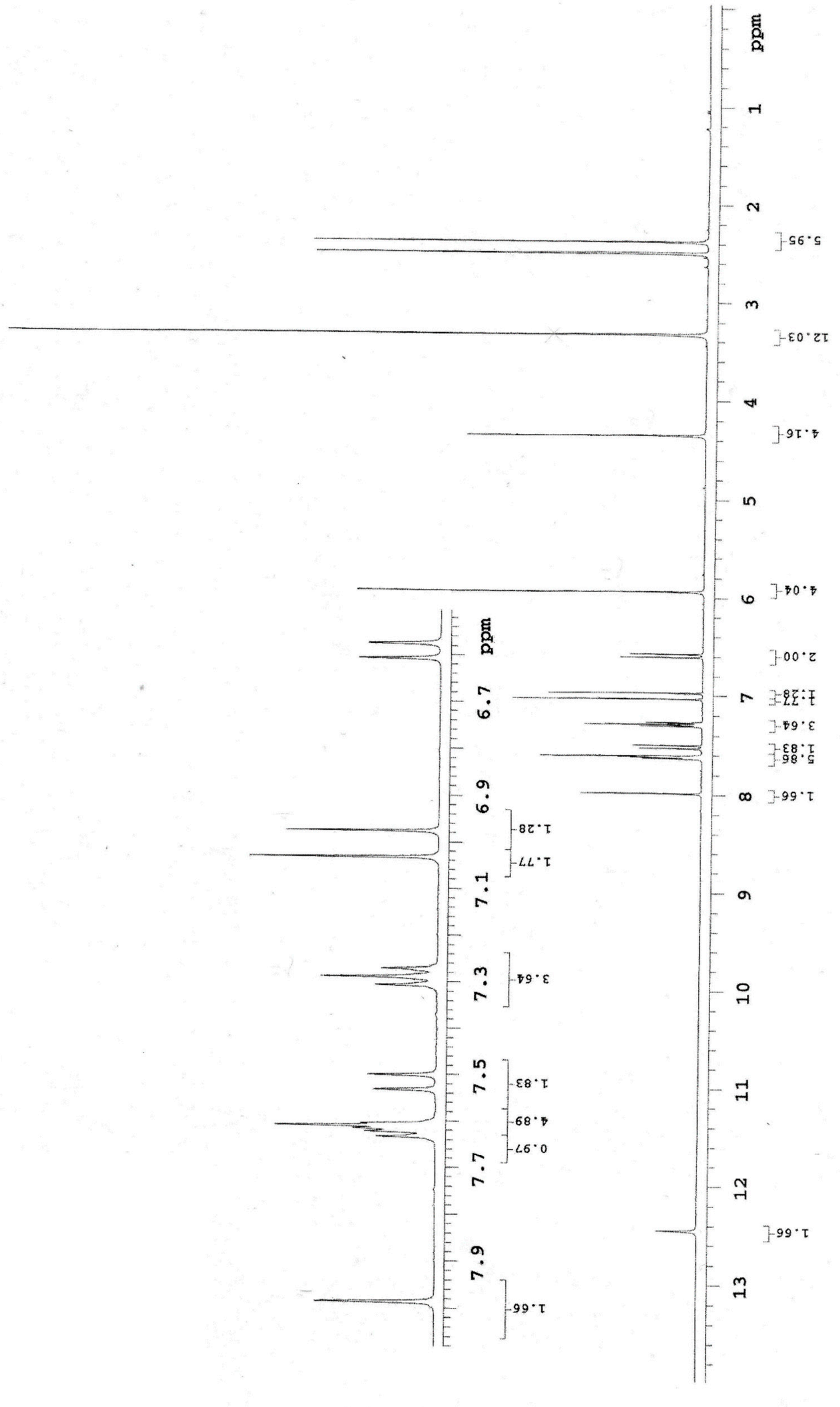

Spectrum 13. ^{1}H NMR of compd **17b** (500 MHz, DMSO-d_6).

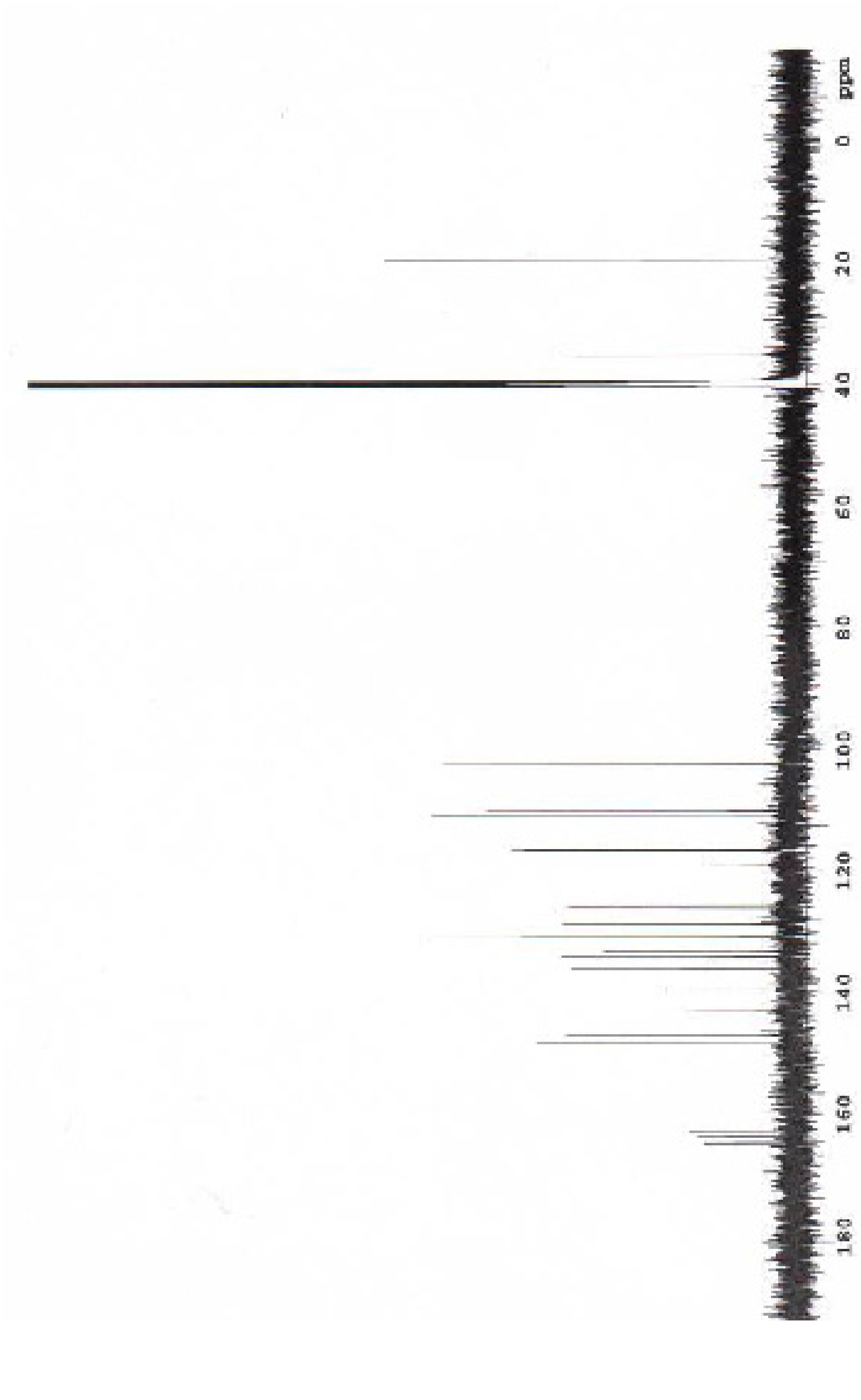

Spectrum 14. ^{13}C NMR of compd **17b** (125 MHz, DMSO-d_6).

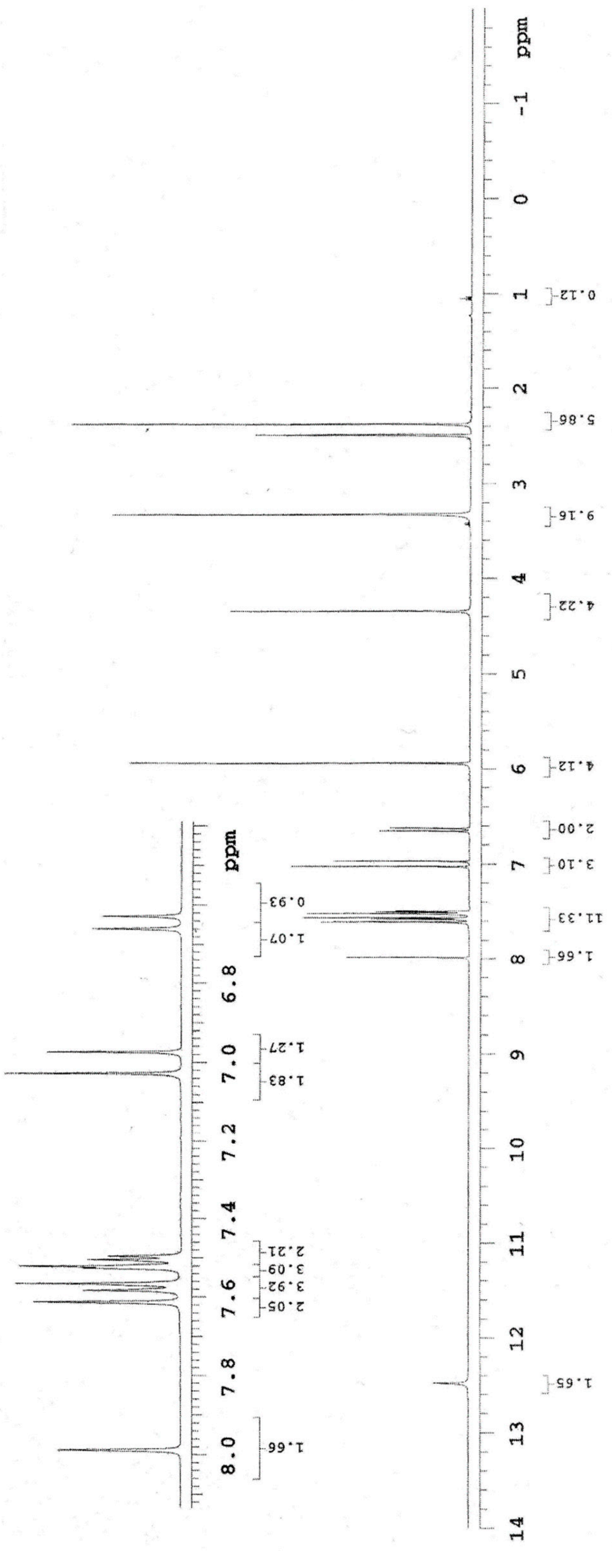

Spectrum 15. ^{1}H NMR of compd **17c** (500 MHz, DMSO-d_6).

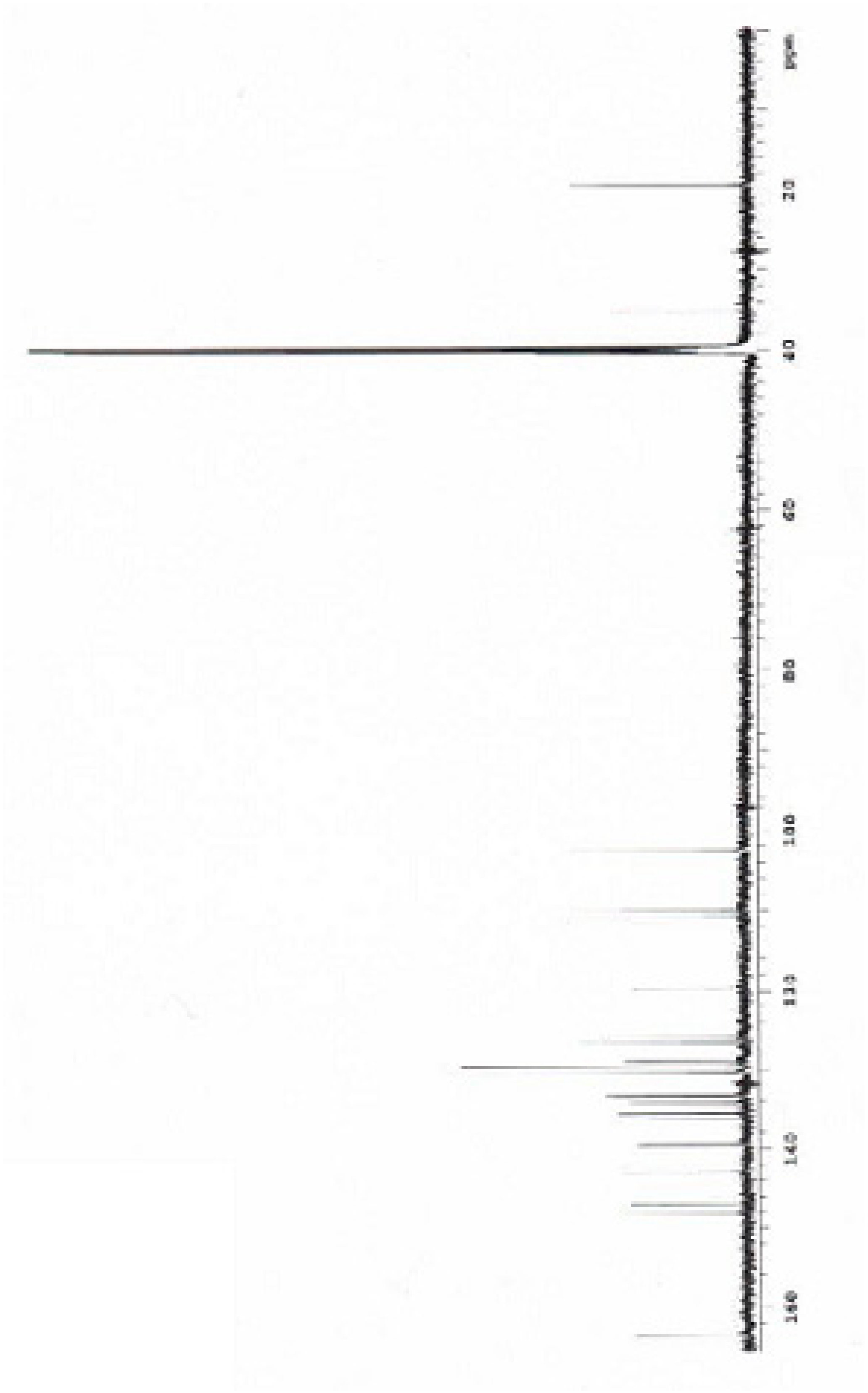

Spectrum 16. ^{13}C NMR of compd **17c** (125 MHz, DMSO-d_6).

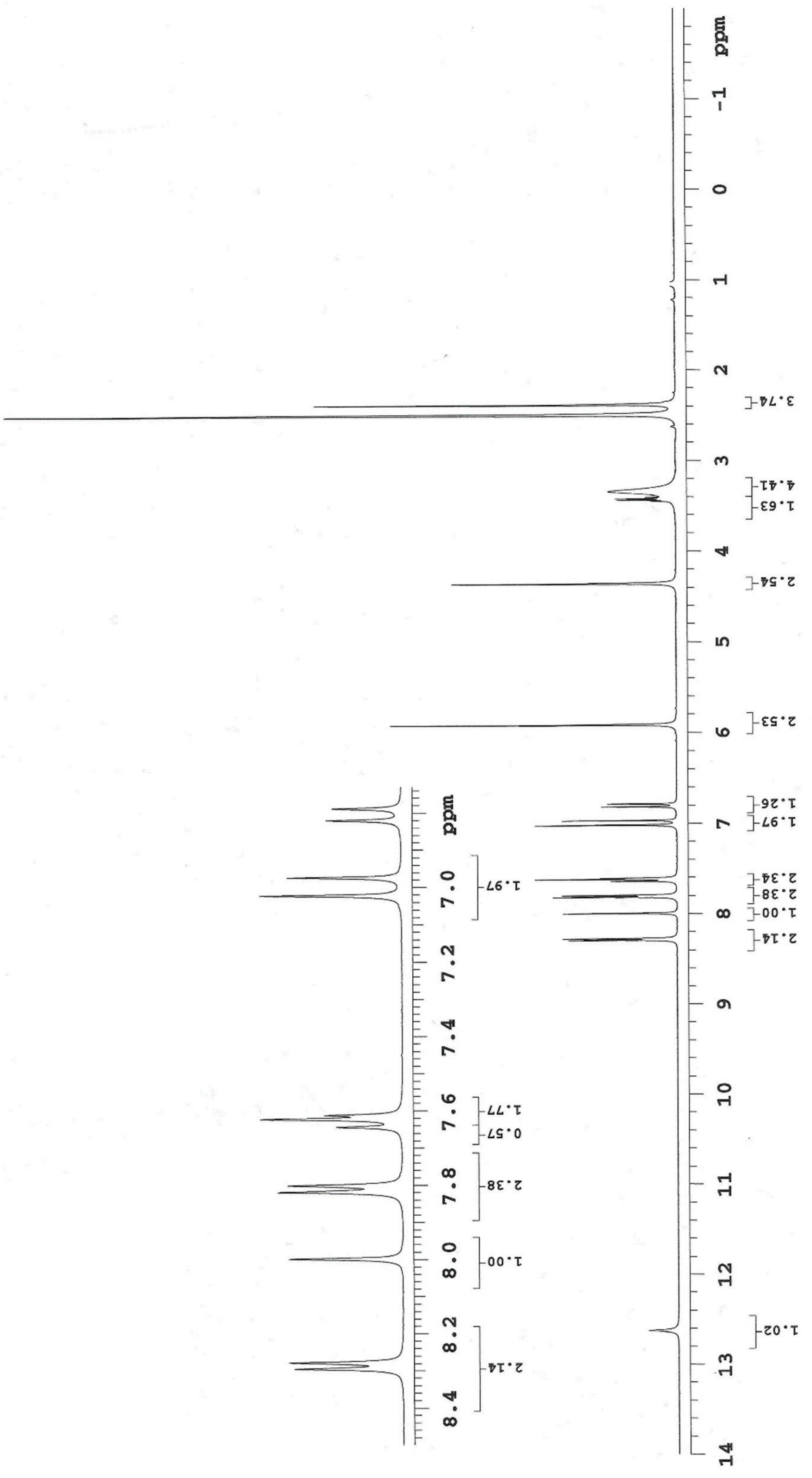

Spectrum 17. ^{1}H NMR of compd **17d** (500 MHz, DMSO-d_6).

Appendix B:

Table S1. Maximum absorbance of the analyzed compounds in 10% DMSO/methanol (*v*/*v*) and in cell culture medium in 0 h and after 96 h.

	**16a**	**16b**	**16c**	**16d**	**16e**	**16f**	**17a**	**17b**	**17c**	**17d**
In 10% DMSO/methanol (*v*/*v*)	315 ± 1	314 ± 0	315 ± 1	304 ± 1	324 ± 0	310 ± 0	315 ± 1	309 ± 1	314 ± 0	324 ± 0
In cell culture medium t_0	300 ± 0	299 ± 1	300 ± 0	296 ± 0	307 ± 1	306 ± 3	300 ± 0	298 ± 0	300 ± 0	305 ± 1
In cell culture medium t_{96}	300 ± 0	300 ± 0	303 ± 1	297 ± 1	306 ± 0	305 ± 2	300 ± 0	298 ± 0	300 ± 0	306 ± 0

The resulting λ_{max} are obtained from three replications and are presented as mean values with the standard deviation (±SD). The concentration of the tested compounds in cell culture medium was 25% (*v*/*v*).

Table S2. Minimum inhibitory concentration [μg/mL] of the cinnamic acid derivatives on MRSA strains.

Compd	MIC [μg/mL]								
	**MRSA13318**	**MRSA13251**	**MRSA12753**	**MRSA2021**	**MRSA21824**	**MRSA15732**	**MRSA12673**	**MRSA21804**	**MRSA12647**
**16a**	2 ± 0.12	2 ± 0.01	2 ± 0.06	2 ± 0.06	1 ± 0.06	2 ± 0.06	2 ± 0.1	4 ± 0.06	4 ± 0.06
**16b**	1 ± 0.06	1 ± 0.06	1 ± 0.06	1 ± 0.06	1 ± 0.06	1 ± 0.06	1 ± 0.1	1 ± 0.12	1 ± 0.12
**16c**	2 ± 0.12	1 ± 0.06	1 ± 0.12	1 ± 0.12	4 ± 0.06	1 ± 0.06	2 ± 0.06	2 ± 0.12	2 ± 0.06
**16d**	1 ± 0.55	1 ± 0.06	1 ± 0.06	1 ± 0.12	1 ± 0.06	1 ± 0.06	2 ± 0.06	2 ± 0.12	2 ± 0.12
**16e**	2 ± 0.06	2 ± 0.05	2 ± 0.06	2 ± 0.06	2 ± 0.06	2 ± 0.06	2 ± 0.06	2 ± 0.06	2 ± 0.12
**16f**	2 ± 0.1	2 ± 0.06	2 ± 0.06	1 ± 0.12	2 ± 0.06	2 ± 0.06	2 ± 0.06	2 ± 0.06	2 ± 0.12
**17a**	1 ± 0.01	1 ± 0.06	1 ± 0.06	1 ± 0.06	1 ± 0.06	1 ± 0.06	8 ± 0.06	8 ± 0.12	8 ± 0.06
**17b**	4 ± 0.12	4 ± 0.06	4 ± 0.06	4 ± 0.06	2 ± 0.06	2 ± 0.06	>125	>125	>125
**17c**	1 ± 0.06	2 ± 0.06	2 ± 0.06	1 ± 0.07	2 ± 0.12	2 ± 0.06	4 ± 0.06	4 ± 0.12	4 ± 0.06
**17d**	4 ± 0.06	4 ± 0.12	4 ± 0.06	4 ± 0.06	4 ± 0.12	4 ± 0.06	4 ± 0.1	4 ± 0.12	4 ± 0.06
**Co-trimoxazole**	<1/0.2	<1/0.2	<1/0.2	<1/0.2	<1/0.2	<1/0.2	<1/0.2	<1/0.2	<1/0.2

Compd	MIC [μg/mL]								
	MRSA18532	MRSA13251	MRSA21791/1	MRSA21791/2	MRSA21791/3	MRSA12677	MRSA6345	MRSAN315 VISA	MRSA56/AS VISA
**16a**	4 ± 0.06	1 ± 0.12	2 ± 0.12	1 ± 0.05	1 ± 0.12	2 ± 0.06	2 ± 0.12	2 ± 0.06	1 ± 0.06
**16b**	2 ± 0.06	1 ± 0.06	1 ± 0.06	1 ± 0.06	1 ± 0.12	2 ± 0.06	1 ± 0.12	4 ± 0.06	1 ± 0.06
**16c**	2 ± 0.06	2 ± 0.06	2 ± 0.06	1 ± 0.05	2 ± 0.12	2 ± 0.06	4 ± 0.06	1 ± 0.12	2 ± 0.06
**16d**	2 ± 0.06	1 ± 0.06	1 ± 0.58	2 ± 0.74	2 ± 0.12	2 ± 0.12	2 ± 0.12	2 ± 0.06	1 ± 0.06
**16e**	4 ± 0.06	1 ± 0.12	2 ± 0.12	1 ± 0.05	2 ± 0.06	2 ± 0.06	2 ± 0.12	1 ± 0.12	1 ± 0.06
**16f**	2 ± 0.06	2 ± 0.12	2 ± 0.06	1 ± 0.12	4 ± 0.06	2 ± 0.06	2 ± 0.06	2 ± 0.06	2 ± 0.06
**17a**	16 ± 0.06	1 ± 0.06	1 ± 0.06	1 ± 0.06	2 ± 0.06	2 ± 0.06	2 ± 0.06	8 ± 0.16	1 ± 0.06
**17b**	>125	2 ± 0.06	2 ± 0.12	1 ± 0.06	4 ± 0.12	8 ± 0.06	4 ± 0.06	>125	2 ± 0.06
**17c**	4 ± 0.06	1 ± 0.1	2 ± 0.06	1 ± 0.12	2 ± 0.06	2 ± 0.12	2 ± 0.12	4 ± 0.06	1 ± 0.12
**17d**	4 ± 0.12	2 ± 0.12	2 ± 0.12	2 ± 0.06	2 ± 0.06	4 ± 0.06	4 ± 0.12	4 ± 0.12	4 ± 0.06
**Co-trimoxazole**	<1/0.2	<1/0.2	<1/0.2	<1/0.2	<1/0.2	<1/0.2	<1/0.2	<1/0.2	<1/0.2

Table S3. Minimum inhibitory concentration [μg/mL] of the cinnamic acid derivatives on MRCNS strains.

Compd	MIC [μg/mL]		
	**MRSE13199**	**MRCNS16248**	**MRCNS15554**
**16a**	1 ± 0.12	2 ± 0.29	2 ± 0.12
**16b**	1 ± 0.12	1 ± 0.12	2 ± 0.29
**16c**	1 ± 0.12	2 ± 0.29	2 ± 0.12
**16d**	1 ± 0.12	2 ± 0.29	1 ± 0.12
**16e**	1 ± 0.12	2 ± 0.29	2 ± 0.12
**16f**	1 ± 0.06	1 ± 0.12	1 ± 0.12
**17a**	1 ± 0.12	2 ± 0.29	2 ± 0.12
**17b**	2 ± 0.29	4 ± 0.12	4 ± 0.06
**17c**	1 ± 0.12	2 ± 0.12	1 ± 0.12
**17d**	2 ± 0.29	4 ± 0.12	4 ± 0.17
**Co-trimoxazole**	<1/0.2	<1/0.2	<1/0.2

Table S4. Minimum inhibitory concentration [µg/mL] of the cinnamic acid derivatives on *Enterococcus* sp. strains.

**Compd**	**MIC [µg/mL]**								
	**Enterococcus sp.3937152**	**Enterococcus sp.3934825**	**Enterococcus sp.12835**	**Enterococcus sp.12848**	**Enterococcus sp.12214**	**Enterococcus sp.12245**	**Enterococcus sp.12961**	**Enterococcus sp.12338**	**Enterococcus sp.16247**
**16a**	4 ± 0.35	4 ± 0.17	2 ± 0.12	2 ± 0.28	4 ± 0.12	4 ± 0.06	4 ± 0.12	4 ± 0.12	2 ± 0.03
**16b**	8 ± 0.12	4 ± 0.12	2 ± 0.12	1 ± 0.06	2 ± 0.12	1 ± 0.06	16 ± 0.06	4 ± 0.12	2 ± 0.12
**16c**	4 ± 0.12	4 ± 0.12	4 ± 0.29	2 ± 0.06	4 ± 0.06	4 ± 0.06	4 ± 0.12	4 ± 0.12	4 ± 0.12
**16d**	4 ± 0.29	8 ± 0.12	4 ± 0.17	2 ± 0.12	2 ± 0.12	2 ± 0.12	8 ± 0.12	2 ± 0.06	1 ± 0.06
**16e**	8 ± 0.06	16 ± 0.12	4 ± 0.12	2 ± 0.06	4 ± 0.12	4 ± 0.12	8 ± 0.06	8 ± 0.12	4 ± 0.06
**16f**	4 ± 0.06	4 ± 0.29	2 ± 0.12	2 ± 0.06	4 ± 0.12	8 ± 0.12	4 ± 0.29	8 ± 0.12	2 ± 0.12
**17a**	16 ± 0.12	16 ± 0.12	8 ± 0.12	2 ± 0.12	4 ± 0.06	8 ± 0.06	32 ± 0.17	8 ± 0.12	2 ± 0.12
**17b**	>125	>125	>125	4 ± 0.12	8 ± 0.12	8 ± 0.06	8 ± 0.12	8 ± 0.12	4 ± 0.06
**17c**	8 ± 0.12	16 ± 0.12	8 ± 0.06	2 ± 0.06	4 ± 0.17	4 ± 0.12	8 ± 0.1	2 ± 0.06	2 ± 0.12
**17d**	>125	32 ± 0.29	16 ± 0.12	4 ± 0.12	8 ± 0.12	8 ± 0.12	8 ± 0.06	8 ± 0.06	4 ± 0.06
**Co-trimoxazole**	>2000/400	>2000/400	2000/400	>2000/400	>2000/400	>2000/400	>2000/400	>2000/400	>2000/400
